# Targeting Raf Kinase Inhibitory Protein Regulation and Function

**DOI:** 10.3390/cancers10090306

**Published:** 2018-09-04

**Authors:** Ali Ekrem Yesilkanal, Marsha Rich Rosner

**Affiliations:** 1 Ben May Department for Cancer Research, University of Chicago, Chicago, IL 60637, USA; aeyesilkanal@gmail.com; 2 Committee on Cancer Biology, University of Chicago, Chicago, IL 60637, USA

**Keywords:** RKIP, metastasis, suppressor, kinase, therapy, signaling

## Abstract

Raf Kinase Inhibitory Protein (RKIP) is a highly conserved kinase inhibitor that functions as a metastasis suppressor in a variety of cancers. Since RKIP can reprogram tumor cells to a non-metastatic state by rewiring kinase networks, elucidating the mechanism by which RKIP acts not only reveals molecular mechanisms that regulate metastasis, but also represents an opportunity to target these signaling networks therapeutically. Although RKIP is often lost during metastatic progression, the mechanism by which this occurs in tumor cells is complex and not well understood. In this review, we summarize our current understanding of RKIP regulation in tumors and consider experimental and computational strategies for recovering or mimicking its function by targeting mediators of metastasis.

## 1. Introduction

Metastasis suppressors are a group of physiological proteins that function to block metastatic progression of tumors. Unlike tumor suppressors, metastasis suppressors do not affect initiation and growth properties of the primary tumors, but rather inhibit the dissemination mechanisms of tumor cells, such as invasion through tissue, intravasation/extravasation, and metastatic colonization. Similar to tumor suppressors, inactivation of metastasis suppressors is a common step in a cancer’s progression into a metastatic disease. This transition can take place by genetic loss of function through deletions or mutations, by epigenetic silencing of the suppressors through DNA methylation and histone modifications, by downregulation of transcriptional machinery controlling the suppressor expression, or by increased rates of post-transcriptional degradation through proteasome and miRNAs. Understanding the details of the regulation and function of metastasis suppressors can create therapeutic opportunities to either restore or mimic the role of these proteins in metastatic cancers. Furthermore, metastasis suppressors and their functional gene networks can be used as prognostic and predictive markers in the clinic for metastatic progression and survival.

Raf kinase inhibitory protein (RKIP), also known as phosphatidylethanolamine binding protein 1 (PEBP1), is a highly conserved protein that is commonly lost or downregulated in cancers. Low RKIP expression correlates very strongly with metastatic phenotype in the majority of cancers, including prostate, breast, pancreatic, lung, cervical cancers and gliomas [1,2,3]. Due to this negative association between RKIP and metastasis, RKIP has been implicated as a powerful predictive biomarker for metastatic risk in patients, as well as a prognostic marker for metastasis-free and overall survival in many solid tumors [1]. Experimentally, over-expressing RKIP blocks invasion in vitro and metastatic progression in vivo [4,5] without affecting growth properties of the primary tumor. RKIP-related gene signatures can be utilized to identify cancer patients at highest risk [6,7,8,9,10]. These clinical and experimental findings establish RKIP as a suppressor of invasion, intravasation, extravasation and metastasis.

At the molecular level, RKIP functions as an inhibitor of kinase activity. It was originally identified as a direct binding partner for Raf kinase [11], and multiple studies have since shown that RKIP interferes with the activation of the Raf-Mek-Erk cascade [12]. Subsequently, it was revealed that it can modulate other signaling pathways such as nuclear factor kappa-light-chain-enhancer of activated B cells (NFκB)-related pathways [13]. RKIP itself can act as a substrate for kinases, and phosphorylation of certain residues can alter RKIP’s binding properties. Most notably, phosphorylation of Ser153 residue by protein kinase C (PKC)switches RKIP’s binding from Raf to G-protein-coupled receptor kinase 2 (GRK2) [14], resulting in activation of protein kinase A (PKA). The mechanism by which this phospho-switch occurs involves a novel salt bridge theft that enables the promotion or disruption of peptide interactions to provide specificity at protein interfaces [15,16]. This illustrates RKIP’s potential role in sensing active signaling networks and modifying the cellular kinome through new protein interactions.

**Figure 1:**
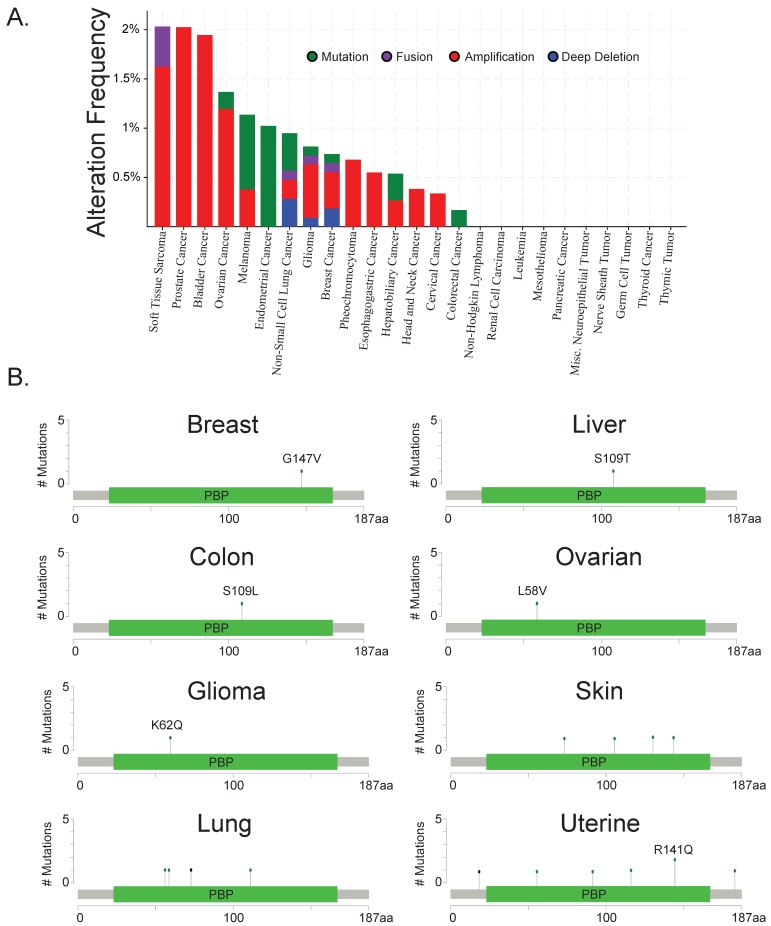
Raf kinase inhibitory protein (RKIP) downregulation in cancers cannot be explained by genetic deletions or mutations as it is rarely deleted or mutated in patient samples (**A**) Frequency of genetic alterations (mutations, fusions, amplifications, and deep deletions) in *PEBP1* (RKIP) genes across multiple TCGA cancer types. (**B**) Residues on *PEBP1* that have been found to be mutated in multiple TCGA cancers. Mutation and sequencing data were accessed through the cBioPortal data base (www.cbioportal.org) [17]. The graphs and figures were generated also on cBioPortal.

RKIP downregulation or total loss is a common event in metastatic solid tumors. However, the mechanism by which tumor cells lose or deactivate RKIP as they become more invasive and metastatic remains elusive. Evidence based on TCGA databases for a variety of cancers suggests that it is usually not a genetic or mutational event (Figure 1A) and only a few mutated residues have been observed (Figure 1B), suggesting transcriptional or post-transcriptional mechanisms are largely responsible for RKIP’s downregulation. Research within the last decade identified promoter methylation and transcriptional inhibition to be one way of silencing RKIP. In addition, multiple groups demonstrated post-transcriptional regulation of RKIP by microRNAs or degradation. Therefore, inducing stable RKIP expression specifically is challenging.

RKIP’s ability to shift tumor cells into a non-metastatic phenotype by reprogramming kinase networks presents a unique opportunity not only to understand molecular mechanisms of metastatic progression, but also to counteract these mechanisms as a therapeutic strategy. In this review, we will cover what is known about mechanisms of RKIP loss in metastatic solid tumors and discuss strategies for recovering its function, with a particular focus on mimicking its downstream effects on metastatic signaling and gene expression.

## 2. Mechanisms of RKIP Downregulation and Strategies for Recovering RKIP Expression

### 2.1. Epigenetic Silencing Through Promoter Methylation and Histone Modifications

Promoter methylation: Promoter methylation is a well-characterized mechanism for epigenetic silencing by which gene transcription is halted through transcription factor inaccessibility, without any genetic alterations. Several studies of tumors, including gastric adenocarcinomas [18,19], esophageal squamous cell carcinomas [20,21], colorectal cancers [22,23], and breast cancers [24], reported that methylation status of the RKIP promoter determined by MSP (methylation specific PCR) strongly correlated with low RKIP expression levels in various advanced stages of the tumors. In gastric and esophageal cancers, carcinoma tissue showed significantly higher incidence rates of hypermethylated RKIP promoters than the adjacent normal mucosal tissue [19,21]. In esophageal cancers, methylated promoter status also correlated with poorly differentiated tumors, as well as lymph node metastases [21]. In agreement with these findings, patients whose tumors had hypermethylated RKIP showed worse overall survival rates compared to unmethylated RKIP cases in gastric cancers [19], implicating RKIP as an independent prognostic marker for survival. In a breast cancer study, however, methylation status of RKIP promoter did not predict survival independently [24], even though it strongly correlated with RKIP mRNA downregulation, which by itself was indicative of poor survival. This finding suggests other potential mechanisms for RKIP loss in advanced disease than just promoter methylation.

Histone modifications: In the prostate cancer cell line DU145, the histone deacetylase (HDAC) inhibitor trichostatin A (TSA) caused a potent increase in RKIP levels [25], raising the possibility that histone deacetylation might play a role in RKIP silencing. Labbozetta et al. similarly reported that, in the triple-negative breast cancer (TNBC) cell line SUM159, histone deacetylase inhibitors induced RKIP mRNA expression in vitro, [26]. By contrast, Lee et al. did not observe induction of RKP mRNA or protein expression by HDAC inhibitors in the triple-negative breast cancer cell lines MDA-MB-231, 1833 (also known as BM1, a bone-tropic subtype of MDA-MB-231 cells), and MDA-MB-436 cells. Rosner and colleagues further noted that BTB domain and CNC homolog 1 (BACH1), a transcription factor which suppresses RKIP transcription, was induced by HDAC inhibitor treatment [27], explaining the lack of RKIP induction by these inhibitors. They also found that the histone methyltransferaseEZH2 (enhancer of zeste 2 polycomb repressive complex 2) interacted with BACH1 in these TNBC cells to inhibit RKIP transcription. Similarly, Yeung and colleagues reported that EZH2 is a negative regulator of RKIP transcription in breast and prostate cancer cells through interaction with Snail [28]. Taken together, these results suggest that epigenetic regulation of RKIP expression by histone acetylation or methylation is a complex process involving multiple factors in a cell- or tissue-dependent manner.

These clinical and experimental findings suggest the use of demethylating agents as a potential strategy for inducing RKIP expression in metastasis cancers. Labbozzetta’s study showed decreased RKIP promoter methylation and increased RKIP mRNA and protein levels in SUM159 cells treated with various doses of the demethylating agent 5-Azacytidine (5-AzaC) [26]. Guo et al. demonstrated similar results in the esophageal cell lines TE-1 and TE-13 [20]. However, Beach et al. showed that 5-AzaC treatment did not induce RKIP expression in the prostate cancer cell line DU145, even though the HDAC inhibitor trichostatin A (TSA) caused a potent increase in RKIP levels [25]. Poma and colleagues observed that 5-AzaC induced RKIP mRNA expression in Hep3B cell line, but the protein levels were not affected. Finally, Rosner and colleagues identified an RKIP-regulated signaling pathway involving an HMGA2 (high mobility group AT-hook 2) - TET1 (ten-eleven translocation 1) - HOX (homeobox gene) axis in the epigenetic regulation of breast cancer cells and showed that 5-AzaC treatment was effective at inhibiting tumor cell invasion by targeting these genes [9]. These results illustrate the cancer type or cell line specific role of RKIP promoter methylation as well as the importance of RKIP-regulated processes that might also be subject to promoter (hydroxy)methylation.

### 2.2. Transcriptional Regulation of RKIP

The role of transcriptional silencing of RKIP in metastatic progression is not well understood. The best characterized transcriptional regulator of RKIP is the epithelial-to-mesenchymal transition (EMT) protein SNAIL. Beach et al. originally identified SNAIL as a direct transcriptional repressor of RKIP in prostate cancer cell lines PC3 and DU145 [25] and demonstrated a negative correlation between SNAIL and RKIP expression levels in publicly available prostate cancer patient data sets. Bonavida and colleagues expanded on this finding by showing that, in prostate and melanoma cell lines, SNAIL expression can be induced by NFκB and YY1 (Yin Yang 1 transcription factor) activity which results in RKIP downregulation [29]. Similarly, Das et al. reported that, in melanoma cell lines, MDA-9 (melanoma differentiation associated gene 9), a PDZ-domain (the postsynaptic density protein (*P*SD-95), discs-large tumor suppressor (*D*lg), and tight junction protein-1 (*Z*O-1) containing scaffold protein that promotes melanoma progression and metastasis, can silence RKIP transcription by activating SNAIL expression through ERK1 and ERK2 (Mitogen-activated protein kinase 3 and 1, respectively) and NFκB signaling [30].

**Figure 2:**
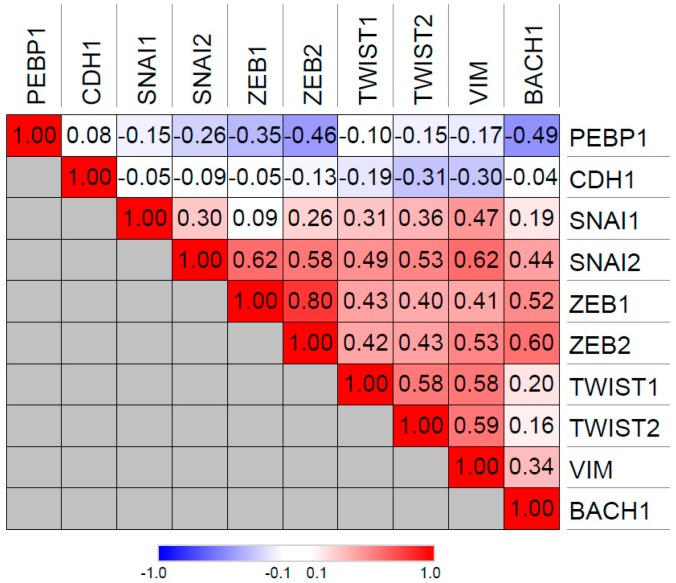
Spearman correlations between *PEBP1* (RKIP), *BACH1* (BTB domain and CNC homolog 1) and the EMT genes *CDH1* (E-cadherin), *SNAI1* (Snail), *SNAI2* (Slug), *ZEB1* (Zinc Finger E-Box Binding Homeobox 1), *ZEB2* (Zinc Finger E-Box Binding Homeobox 2), *TWIST1* (Twist Family BHLH Transcription Factor 1), *TWIST2* (Twist Family BHLH Transcription Factor 2), and *VIM* (vimentin) gene expression in TCGA breast cancer cohort. Correlations are based on RNA-seq data, accessed through cBioPortal database.

BACH1 similarly inhibits RKIP transcription in TNBCs [10,27] and promotes TNBC metastasis [10,31]. In contrast to Snail (*SNAI1*), BACH1 expression is significantly inversely correlated with RKIP expression in the TCGA breast cancer patient database, suggesting that BACH1 may be the main negative regulator of RKIP in breast cancer (Figure 2). Like Snail, BACH1 positively correlates with expression of EMT-associated genes such as vimentin and ZEB1/2 (Zinc Finger E-Box Binding Homeobox 1/2), implying a role in the epithelial-mesenchymal transition (Figure 2) Interestingly, both Snail and BACH1 negatively regulate their own promoters, indicative of a negative feedback loop that limits expression of these two transcription factors. Furthermore, both Snail and BACH1 are downstream targets of RKIP as well as negative regulators of RKIP [27]. Lee et al. showed that this relationship represents a potential bi-stable state that could be stochastically disrupted to switch cells from an RKIP-expressing, non-metastatic mode to an RKIP-depleted, BACH1-expressing mode, driving cells to metastasis through a nongenetic mechanism [27].

RKIP expression may also be regulated by other factors. In a non-tumorigenic immortalized prostate cell line RWPE-1, Zhang et al. reported that androgen receptor can directly bind to the RKIP promoter and activate RKIP transcription upon dihydrotestosterone treatment [32]. In addition, another study showed that transcriptional regulators Sp1 (Specificity Protein 1), CREB (CAMP Responsive Element Binding Protein), and p300 (E1A Binding Protein P300) can bind and positively regulate RKIP expression in A375 melanoma and HeLa cells. [33]. Whether these particular transcription factors are associated with metastatic disease in melanoma or ovarian cancer has not been established.

### 2.3. Post-Transcriptional Regulation of RKIP

As an alternative mechanism for RKIP silencing, several microRNAs have been identified in recent years as inhibitors of RKIP expression in cancers. Li et al. demonstrated that miR-27a inhibits RKIP mRNA levels in lung adenocarcinomas, resulting in cisplatin resistance [34]. In prostate cancers, miR-543 [35] and miR-23a [36] have been shown to target RKIP. The same study demonstrated that the tumor suppressor long non-coding RNA XIST asserts its suppressive function by inhibiting miR-23a and keeping RKIP expression levels high in prostate cancers [36]. Another study identified RKIP as a miR-23a target in acute myelogenous leukemia (AML) patients as well [37]. In breast cancer cell lines, RKIP is targeted by miR-224, and inhibition of miR-224 mimics RKIP’s anti-invasive function and RKIP-mediated gene transcription [38]. However, regulation of RKIP by microRNAs might be cancer type specific, as Poma et al. reported that miR-224 does not inhibit RKIP in hepatocellular carcinoma cell lines [39]. Despite the promise of microRNAs as therapeutic targets for activation of RKIP, translating them into actionable drugs in the clinic has been technically challenging.

Protein degradation is the least explored mode of RKIP regulation. Kim et al. demonstrated that, in hepatocellular carcinomas, the kinase inhibitor Sorafenib promoted RKIP degradation and induced ERK activation as a drug resistance mechanism [40]. Moen et al. showed that H. pylori preferentially phosphorylates RKIP at S153 to drive it to the nucleus and promote cell apoptosis while simultaneously targeting unphosphorylated S153 RKIP for proteasome-mediated degradation in gastric cancer cells [41]. Wen and colleagues showed that CDK5 (Cyclin Dependent Kinase 5)-mediated phosphorylation of RKIP on T42 residue triggers recruitment of the chaperone molecule Hsc-70 and subsequent autophagy-mediated degradation of RKIP in Parkinson disease [42]. RKIP silencing by post-translational mechanisms is a largely unknown field that needs to be explored in different cell or tissue types. Without identifying relevant mechanisms to target RKIP for degradation, it will be difficult to take advantage of this regulatory mechanism therapeutically.

### 2.4. Chemical Induction of RKIP

Nitric oxide is an organic molecule that can induce RKIP expression [43,44]. Bonavida and colleagues have shown that DETANONOate, a nitric oxide donor, functions as an inhibitor of NFκB signaling and works to lift SNAIL-mediated transcriptional suppression on RKIP promoter [43]. This reverses EMT in prostate cell lines and xenograft models. DETANONOate-induced RKIP expression also sensitizes resistant cancer cells to apoptotic agents such as TRAIL (TNF-related apoptosis-inducing ligand) and CDDP (cisplatin).

In certain cases, chemo-/immuno-/radio-therapy can also induce RKIP expression, which is important for the cytotoxic action of these therapeutic agents. Gemcitibine, an inhibitor of DNA synthesis and cell cycle, induces RKIP expression and synergizes with Sorafenib (a small molecule inhibitor targeting Vascular Endothelial Growth Factor Reeptor (VEGFR), Platelet-derived Growth Factor Receptor (PDGFR), and Raf) in non-small cell lung cancer (NSCLC) [45] and in pancreatic cancer [46] cell lines to block proliferation and increase apoptosis. The Epidermal Growth Factor Receptor (EGFR) inhibitor Erlotinib also showed synergistic effect with Sorafenib and increased RKIP expression in lung cancers [47]. 9NC (a topoisomerase I inhibitor) in prostate and breast cell lines [48], anti-mitotic agents ENMD-1198 and MKC-1 in prostate cancer cell lines [49], have all been shown to exert their effect through upregulation of RKIP. One study revealed that the bacterial antibiotic Gemifloxacin can also block migration and invasion of breast cancer cell lines MDA-MB-231 and MB-453 through RKIP protein induction [50]. Conversely, locostatin, a migration inhibitor, has been reported to act through specific inhibition of RKIP association with Raf [51,52]. However, studies by Shemon et al. showed that locostatin acts in a nonspecific manner independent of RKIP/Raf to inhibit cell migration and cause cytoskeletal defects [53]. One caution in all of these studies is the high degree of off-target effects of drugs.

### 2.5. Regulation and Function of RKIP in Liquid Cancers

Clinical implications of RKIP expression in liquid cancers, reviewed in detail by Lamiman et al. [1], is a field that requires more extensive investigation. Although the molecular networks regulated by RKIP seem to be similar in liquid and solid tumors, the clinical outcome of RKIP expression can differ. Consistent with its inhibition of the Raf-MEK-ERK cascade, RKIP-overexpression in hematopoietic cells impaired the oncogenic activity of RAS (rat sarcoma) kinase in proliferation and soft agar colonization assays. Similarly, RKIP expression is lost in 19 of 103 acute myeloid leukemia (AML) patients, suggesting a potential role in AML progression for some individuals [54]. In a subtype of AML called myeloid sarcoma, leukemia cells can leave the blood system and form tumors in solid tissue by a process similar to extravasation and metastasis of solid cancers. Caraffini et al. demonstrated that RKIP functions as a suppressor of this metastatic process [55]. Knocking down RKIP in AML cell lines resulted in more cells infiltrating the tissue and forming tumors in a chorioallantoic membrane assay, whereas RKIP over-expression showed the opposite effect. In a small group of AML patients, RKIP loss was enriched in myeloid sarcoma cases, and occurred at both protein and mRNA levels. The authors speculated that the loss of RKIP in these patients is probably through miR-23a, as they have previously demonstrated negative regulation of RKIP expression by miR-23a in AML patients [37]. It should also be noted that RKIP loss in AML patients correlates with mutations in RAS, arguing for a potential role for mutant RAS in RKIP regulation [54].

Despite its inhibitory effect on extravasation and tissue infiltration in myeloid sarcoma, loss of RKIP seems to be a marker for good prognosis in AML [54], contrary to solid tumors. Patients whose cancer lacked RKIP expression showed longer relapse-free and overall survival rates upon standard chemotherapy. This could be attributed, at least in part, to the differences in mutational, epigenetic and microenvironmental landscape of blood cancers versus solid cancers. RKIP reprograms the tumor microenvironment to inhibit metastasis (e.g., by blocking tumor-associated macrophage recruitment [7]), which is vastly different in liquid tumors. Liquid cancers also tend to have mutations in global epigenetic regulators such as DNMT (DNA methyl-transferases) and TET (Ten-Eleven Translocation) genes (reviewed in [56]), which contribute to phenotypic output with or without anti-cancer treatment. Interestingly, the same TET-HOX signaling pathway promotes AML [57] but suppresses breast cancer [9], suggesting that the internal cell signaling environment differs significantly between solid and liquid tumors. Alternatively, the fact that RKIP has different functions dependent on its S153 phosphorylation state, may play a role in different cell-specific outcomes. For example, in multiple myeloma, RKIP overexpression is common, but in half the cases RKIP is found in its p-S153 form [58], which confounds prognostic and predictive analyses. Thus, post-translational modifications of the RKIP protein, such as phosphorylation of the S153 residue that activates both ERK and PKA kinases, might also contribute to the differences in RKIP function as well as its prognostic efficacy.

In non-Hodgkin’s lymphoma B cells, Rituximab, an FDA-approved chimeric anti-human CD20 monoclonal antibody, sensitizes resistant tumor cells to drug treatment by inducing RKIP expression leading to down-regulation of ERK phosphorylation and the anti-apoptotic gene Bcl-XL (B-cell lymphoma–extra large) [59]. This is similar to RKIP’s chemo-sensitization role in prostate cancer (previously discussed). Two other monoclonal antibodies targeting CD20, BM-ca and LFB-R603 showed the same results [60,61].

Taken together, these studies demonstrate that it is important to take into account specific cell types when assessing the association of RKIP with clinical outcome. Particularly when comparing liquid versus solid tumors, the impact of the different microenvironments as well as the unique intracellular regulators on tumor cell gene expression and signaling could lead to different mechanisms for regulating RKIP at multiple levels, including epigenetic/transcriptional, translational, and post-translational.

## 3. Identifying Downstream Networks to Mimic RKIP Function

Inhibiting mechanisms that suppress RKIP transcription is a viable strategy to reactivate RKIP function in metastatic cancers. As discussed above, this method could revert the invasive phenotype in certain cancers. However, the presence of post-transcriptional and post-translational mechanisms to downregulate RKIP as well as the possibility of off-target effects may limit the success of these approaches. An alternative approach is to elucidate downstream kinase and gene networks regulated by RKIP and mimic its molecular and cellular function therapeutically. Since RKIP is an inhibitor of kinases across metastatic cancers, identifying its kinase targets can reveal key signaling mechanisms of invasion and metastasis that are targetable with small molecule inhibitors. Kinomic and transcriptomic reprogramming by RKIP in cancers can be used as a guide to develop anti-metastatic therapies and build gene signatures for precision medicine.

### 3.1. Kinase Targets

RKIP regulates the activity of multiple kinase pathways that are important for metastasis. As an inhibitor of the Raf-Mek-Erk cascade, RKIP binds to Raf-1 and blocks activation of downstream mitogen-activated protein kinases (MAPKs) [11,62,63]. RKIP was reported to inhibit NFκB signaling by directly binding to NIK (NFκB inducing kinase) and TAK1 (transforming growth factor beta (TGFB)-activated kinase 1) [13,64]. RKIP over-expression can also reduce SRC and FAK (focal adhesion kinase) activation under certain conditions [29,48]. All of these signaling pathways play crucial roles in cell motility/invasion and metastasis.

Pharmacological inhibition of these pathways can potentially mimic RKIP. Raf and Mek inhibitors have been extensively studied for their anti-tumorigenic and anti-metastatic activities and are in clinical trials for metastatic cancers [65]. Inhibition of NFκB signaling pathway with IKK inhibitors [66] or by NFκB inhibitors [67], for example, has an anti-metastatic effect on tumor progression. FAK inhibitors can block metastasis in multiple cancers by inhibiting cell motility as well as remodeling the tumor microenvironment and sensitizing tumors to cytotoxic treatments [68]. Inhibition of Src kinase yields similar results in pre-clinical studies [69]. However, this therapeutic approach is complicated by the fact that that tumors usually become resistant to kinase inhibitors by activating alternative feedback mechanisms [70].

### 3.2. Mimicking Microenvironmental Effects of RKIP in Tumors

RKIP’s anti-metastatic function is at least partially mediated through its effect on the tumor microenvironment. Rosner and colleagues demonstrated that RKIP over-expression in metastatic TNBC mouse models robustly blocks recruitment of tumor-associated macrophages (TAMs) into the tumors through a mechanism dependent upon the chemokine CCL5 (C-C motif chemokine ligand 5) [7]. TAMs are known to be critical components of metastatic progression in solid tumors, as macrophages can enhance tumor cell invasion and intravasation, and promote tumor angiogenesis [71].

More broadly, using species-specific RNA sequencing in a xenograft TNBC mouse model, Bainer et al. demonstrated that gene expression in metastatic breast tumors is widely correlated with gene expression in local stroma of both mouse xenografts and human patients [6]. Moreover, changes in stromal gene expression elicited by tumors that do or do not express RKIP is a better predictor of breast cancer subtype and patient metastasis-free survival than tumor gene expression. These findings show that understanding microenvironmental functions of RKIP can open new prognostic and therapeutic avenues.

### 3.3. Computational Approaches to Build Actionable RKIP Gene Networks

Computational strategies are extremely useful in identifying critical signaling pathways and targetable factors, especially when powered by patient-derived data. Using known biological interactions (or experimentally-derived interactions) as filters is a powerful method while building networks because it reduces noise [72]. RKIP, as a selective metastasis suppressor, is a potent filter for generating statistical correlations focused on the metastatic process.

To systemically identify downstream mediators of RKIP, Rosner and colleagues have employed integrated approaches that combine statistical/computational analysis of breast cancer gene expression data and experimentally-validated gene interactions. Dangi-Garimella et al. first experimentally identified a pro-metastatic signaling cascade involving Myc/LIN28/let-7 as an RKIP target [4]. Then, Yun et al. used this RKIP/let-7 interaction as a computational filter to connect the RKIP signaling cascade to previously-described bone metastasis signature genes [10,30]. Specifically, they used statistical and machine learning approaches involving gene set analysis (GSA) and Random Forest (RF) on a compilation of publicly-available microarray gene expression data from patient tumors. With this method, they identified HGMA2 and BACH1 as likely mediators that connect RKIP signaling to downstream bone metastasis genes MMP1 (matrix metalloproteinase 1), OPN (osteopontin), and CXCR4 (chemokine C-X-C motif receptor 4). Yun et al. then validated down-regulation of HMGA2 and BACH1 by RKIP and let-7 experimentally in vitro and in vivo. Thus, these studies used patient gene expression data to generate hypothetical signaling networks that were then validated experimentally. The key biomarkers identified can also serve as therapeutic targets to mimic the anti-metastatic action of RKIP.

Exploring and expanding RKIP-regulated gene networks using patient-derived expression data also allow for developing predictive and prognostic gene signatures that have mechanistic relevance to specific patient cohorts. After identifying RKIP-regulated signaling networks in tumors and their microenvironment, the Rosner group generated several RKIP pathway-based gene signatures for predicting metastasis-free survival of breast cancer patients [6,7,8,9,10]. The biomarkers used in these gene signatures span a wide range of metastatic processes such as extracellular matrix remodeling, epigenetic regulation, tumor-associated macrophage recruitment, and reprogramming of stromal gene expression by tumors. While these signatures facilitate the identification of patients who would benefit from RKIP-like regulation, the challenge has been to come up with therapeutic treatments that either mimic or reactivate RKIP functionally in tumor cells.

With the completion of TCGA and ease of access to clinical sequencing data from multiple studies, building clinically relevant hypothetical gene networks and signatures is becoming less challenging. Gene signatures built on experimentally validated mechanistic gene-gene interactions can (1) help identify/stratify patients for guided/personalized treatment, (2) be used as biomarkers for predicting drug efficacy or sensitivity in patients, and (3) be combined with machine learning methods for drug discovery or drug repurposing for a particular biological function. Conceptually, novel anti-metastatic therapies can be discovered as long as the therapeutic approach being tested is able to target genes in patients identified as high risk by RKIP-related gene signatures. In parallel, therapeutic treatments that mimic RKIP’s anti-metastatic functions would be more effective in patients who are at high risk for metastasis based on their RKIP pathway signature score.

## 4. Conclusions

In sum, RKIP is an important suppressor of metastasis that would be most effective if induced in metastatic cells. However, even in the absence of RKIP protein, it is possible to leverage RKIP targets and patient data to identify novel targets such as specific kinases that, if inhibited together, can mimic the anti-metastatic properties of RKIP and make tumors more homogeneous and more susceptible to conventional cytotoxic therapy.

## References

[1] Lamiman K., Keller J.M., Mizokami A., Zhang J., Keller E.T. (2014). Survey of Raf kinase inhibitor protein (RKIP) in multiple cancer types. Crit. Rev. Oncog..

[2] Martinho O., Granja S., Jaraquemada T., Caeiro C., Miranda-Gonçalves V., Honavar M., Costa P., Damasceno M., Rosner M.R., Lopes J.M. (2012). Downregulation of RKIP is associated with poor outcome and malignant progression in gliomas. PLoS ONE.

[3] Martinho O., Pinto F., Granja S., Miranda-Gonçalves V., Moreira M.A.R., Ribeiro L.F.J., di Loreto C., Rosner M.R., Longatto-Filho A., Reis R.M. (2013). RKIP inhibition in cervical cancer is associated with higher tumor aggressive behavior and resistance to cisplatin therapy. PLoS ONE.

[4] Dangi-Garimella S., Yun J., Eves E.M., Newman M., Erkeland S.J., Hammond S.M., Minn A.J., Rosner M.R. (2009). Raf kinase inhibitory protein suppresses a metastasis signalling cascade involving LIN28 and let-7. EMBO J..

[5] Fu Z., Smith P.C., Zhang L., Rubin M.A., Dunn R.L., Yao Z., Keller E.T. (2003). Effects of raf kinase inhibitor protein expression on suppression of prostate cancer metastasis. J. Natl. Cancer Inst..

[6] Bainer R., Frankenberger C., Rabe D., An G., Gilad Y., Rosner M.R. (2016). Gene expression in local stroma reflects breast tumor states and predicts patient outcome. Sci. Rep..

[7] Frankenberger C., Rabe D., Bainer R., Sankarasharma D., Chada K., Krausz T., Gilad Y., Becker L., Rosner M.R. (2015). Metastasis Suppressors Regulate the Tumor Microenvironment by Blocking Recruitment of Prometastatic Tumor-Associated Macrophages. Cancer Res..

[8] Lee U., Frankenberger C., Yun J., Bevilacqua E., Caldas C., Chin S.-F., Rueda O.M., Reinitz J., Rosner M.R. (2013). A prognostic gene signature for metastasis-free survival of triple negative breast cancer patients. PLoS ONE.

[9] Sun M., Song C.-X., Huang H., Frankenberger C.A., Sankarasharma D., Gomes S., Chen P., Chen J., Chada K.K., He C. (2013). HMGA2/TET1/HOXA9 signaling pathway regulates breast cancer growth and metastasis. Proc. Natl. Acad. Sci. USA.

[10] Yun J., Frankenberger C.A., Kuo W.-L., Boelens M.C., Eves E.M., Cheng N., Liang H., Li W.-H., Ishwaran H., Minn A.J. (2011). Signalling pathway for RKIP and Let-7 regulates and predicts metastatic breast cancer. EMBO J..

[11] Yeung K., Seitz T., Li S., Janosch P., McFerran B., Kaiser C., Fee F., Katsanakis K.D., Rose D.W., Mischak H. (1999). Suppression of Raf-1 kinase activity and MAP kinase signalling by RKIP. Nature.

[12] Yesilkanal A.E., Rosner M.R. (2014). Raf kinase inhibitory protein (RKIP) as a metastasis suppressor: Regulation of signaling networks in cancer. Crit. Rev. Oncog..

[13] Zhao J., Wenzel S. (2014). Interactions of RKIP with inflammatory signaling pathways. Crit. Rev. Oncog..

[14] Lorenz K., Lohse M.J., Quitterer U. (2003). Protein kinase C switches the Raf kinase inhibitor from Raf-1 to GRK-2. Nature.

[15] Skinner J.J., Rosner M.R. (2014). RKIP Structure Drives Its Function: A Three-State Model for Regulation of RKIP. Crit. Rev. Oncog..

[16] Skinner J.J., Wang S., Lee J., Ong C., Sommese R., Sivaramakrishnan S., Koelmel W., Hirschbeck M., Schindelin H., Kisker C. (2017). Conserved salt-bridge competition triggered by phosphorylation regulates the protein interactome. Proc. Natl. Acad. Sci. USA..

[17] Gao J., Aksoy B.A., Dogrusoz U., Dresdner G., Gross B., Sumer S.O., Sun Y., Jacobsen A., Sinha R., Larsson E. (2013). Integrative analysis of complex cancer genomics and clinical profiles using the cBioPortal. Sci. Signal..

[18] Guo W., Dong Z., Guo Y., Lin X., Chen Z., Kuang G., Yang Z. (2013). Aberrant methylation and loss expression of RKIP is associated with tumor progression and poor prognosis in gastric cardia adenocarcinoma. Clin. Exp. Metastasis.

[19] LI D.-X., CAI H.-Y., WANG X., FENG Y.-L., CAI S.-W. (2014). Promoter methylation of Raf kinase inhibitory protein: A significant prognostic indicator for patients with gastric adenocarcinoma. Exp. Ther. Med..

[20] Guo W., Dong Z., Lin X., Zhang M., Kuang G., Zhu T. (2012). Decreased expression and aberrant methylation of Raf kinase inhibitory protein gene in esophageal squamous cell carcinoma. Cancer Investig..

[21] Wei H., Liu Z., She H., Liu B., Gu J., Wei D., Zhang X., Wang J., Qi S., Ping F. (2017). Promoter methylation and expression of Raf kinase inhibitory protein in esophageal squamous cell carcinoma. Oncol. Lett..

[22] Al-Mulla F., Hagan S., Al-Ali W., Jacob S.P., Behbehani A.I., Bitar M.S., Dallol A., Kolch W. (2008). Raf kinase inhibitor protein: mechanism of loss of expression and association with genomic instability. J. Clin. Pathol..

[23] Minoo P., Baker K., Goswami R., Chong G., Foulkes W.D., Ruszkiewicz A.R., Barker M., Buchanan D., Young J., Jass J.R. (2006). Extensive DNA methylation in normal colorectal mucosa in hyperplastic polyposis. Gut..

[24] Kim G.-E., Kim N.I., Lee J.S., Park M.H., Yoon J.H. (2017). Reduced RKIP Expression is Associated with Breast Neoplastic Progression and is Correlated with Poor Outcomes and Aberrant Methylation in Breast Carcinoma. Appl. Immunohistochem. Mol. Morphol. AIMM.

[25] Beach S., Tang H., Park S., Dhillon A.S., Keller E.T., Kolch W., Yeung K.C. (2008). Snail is a repressor of RKIP transcription in metastatic prostate cancer cells. Oncogene.

[26] Labbozzetta M., Poma P., Vivona N., Gulino A., D’Alessandro N., Notarbartolo M. (2015). Epigenetic changes and nuclear factor-κB activation, but not microRNA-224, downregulate Raf-1 kinase inhibitor protein in triple-negative breast cancer SUM 159 cells. Oncol. Lett..

[27] Lee J., Lee J., Farquhar K.S., Yun J., Frankenberger C.A., Bevilacqua E., Yeung K., Kim E.-J., Balázsi G., Rosner M.R. (2014). Network of mutually repressive metastasis regulators can promote cell heterogeneity and metastatic transitions. Proc. Natl. Acad. Sci. USA.

[28] Ren G., Baritaki S., Marathe H., Feng J., Park S., Beach S., Bazeley P.S., Beshir A.B., Fenteany G., Mehra R. (2012). Polycomb protein EZH2 regulates tumor invasion via the transcriptional repression of the metastasis suppressor RKIP in breast and prostate cancer. Cancer Res..

[29] Bonavida B., Baritaki S. (2011). Dual role of NO donors in the reversal of tumor cell resistance and EMT: Downregulation of the NF-κB/Snail/YY1/RKIP circuitry. Nitric Oxide Biol. Chem..

[30] Das S.K., Bhutia S.K., Sokhi U.K., Azab B., Su Z., Boukerche H., Anwar T., Moen E.L., Chatterjee D., Pellecchia M. (2012). Raf kinase inhibitor RKIP inhibits MDA-9/syntenin-mediated metastasis in melanoma. Cancer Res..

[31] Liang Y., Wu H., Lei R., Chong R.A., Wei Y., Lu X., Tagkopoulos I., Kung S.-Y., Yang Q., Hu G. (2012). Transcriptional network analysis identifies BACH1 as a master regulator of breast cancer bone metastasis. J. Biol. Chem..

[32] Zhang H., Wu J., Keller J.M., Yeung K., Keller E.T., Fu Z. (2012). Transcriptional regulation of RKIP expression by androgen in prostate cells. Cell Physiol. Biochem. Int. J. Exp. Cell Physiol. Biochem. Pharmacol..

[33] Zhang B., Wang O., Qin J., Liu S., Sun S., Liu H., Kuang J., Jiang G., Zhang W. (2013). *cis*-Acting elements and trans-acting factors in the transcriptional regulation of raf kinase inhibitory protein expression. PLoS ONE.

[34] Li J., Wang Y., Song Y., Fu Z., Yu W. (2014). miR-27a regulates cisplatin resistance and metastasis by targeting RKIP in human lung adenocarcinoma cells. Mol. Cancer.

[35] Du Y., Liu X.-H., Zhu H.-C., Wang L., Ning J.-Z., Xiao C.-C. (2017). MiR-543 Promotes Proliferation and Epithelial-Mesenchymal Transition in Prostate Cancer via Targeting RKIP. Cell Physiol. Biochem. Int. J. Exp. Cell Physiol. Biochem. Pharmacol..

[36] Du Y., Weng X.-D., Wang L., Liu X.-H., Zhu H.-C., Guo J., Ning J.-Z., Xiao C.-C. (2017). LncRNA XIST acts as a tumor suppressor in prostate cancer through sponging miR-23a to modulate RKIP expression. Oncotarget.

[37] Hatzl S., Geiger O., Kuepper M.K., Caraffini V., Seime T., Furlan T., Nussbaumer E., Wieser R., Pichler M., Scheideler M. (2016). Increased Expression of miR-23a Mediates a Loss of Expression in the RAF Kinase Inhibitor Protein RKIP. Cancer Res..

[38] Huang L., Dai T., Lin X., Zhao X., Chen X., Wang C., Li X., Shen H., Wang X. (2012). MicroRNA-224 targets RKIP to control cell invasion and expression of metastasis genes in human breast cancer cells. Biochem. Biophys. Res. Commun..

[39] Poma P., Labbozzetta M., Vivona N., Porcasi R., D’Alessandro N., Notarbartolo M. (2012). Analysis of possible mechanisms accounting for raf-1 kinase inhibitor protein downregulation in hepatocellular carcinoma. Omics J. Integr. Biol..

[40] Kim J.S., Choi G.H., Jung Y., Kim K.M., Jang S.-J., Yu E.S., Lee H.C. (2018). Downregulation of Raf-1 kinase inhibitory protein as a sorafenib resistance mechanism in hepatocellular carcinoma cell lines. J. Cancer Res. Clin. Oncol..

[41] Moen E.L., Wen S., Anwar T., Cross-Knorr S., Brilliant K., Birnbaum F., Rahaman S., Sedivy J.M., Moss S.F., Chatterjee D. (2012). Regulation of RKIP function by Helicobacter pylori in gastric cancer. PLoS ONE.

[42] Wen Z., Shu Y., Gao C., Wang X., Qi G., Zhang P., Li M., Shi J., Tian B. (2014). CDK5-mediated phosphorylation and autophagy of RKIP regulate neuronal death in Parkinson’s disease. Neurobiol. Aging.

[43] Baritaki S., Huerta-Yepez S., Sahakyan A., Karagiannides I., Bakirtzi K., Jazirehi A., Bonavida B. (2010). Mechanisms of nitric oxide-mediated inhibition of EMT in cancer: Inhibition of the metastasis-inducer Snail and induction of the metastasis-suppressor RKIP. Cell Cycle.

[44] Tsao D.-A., Yu H.-S., Chang H.-R. (2009). Nitric oxide enhances expression of raf kinase inhibitor protein in keratinocytes. Exp. Dermatol..

[45] Pasqualetti G., Ricciardi S., Mey V., Del Tacca M., Danesi R. (2011). Synergistic cytotoxicity, inhibition of signal transduction pathways and pharmacogenetics of sorafenib and gemcitabine in human NSCLC cell lines. Lung Cancer Amst. Neth..

[46] Ricciardi S., Mey V., Nannizzi S., Pasqualetti G., Crea F., Del Tacca M., Danesi R. (2010). Synergistic cytotoxicity and molecular interaction on drug targets of sorafenib and gemcitabine in human pancreas cancer cells. Chemotherapy.

[47] Giovannetti E., Labots M., Dekker H., Galvani E., Lind J.S.W., Sciarrillo R., Honeywell R., Smit E.F., Verheul H.M., Peters G.J. (2013). Molecular mechanisms and modulation of key pathways underlying the synergistic interaction of sorafenib with erlotinib in non-small-cell-lung cancer (NSCLC) cells. Curr. Pharm. Des..

[48] Chatterjee D., Bai Y., Wang Z., Beach S., Mott S., Roy R., Braastad C., Sun Y., Mukhopadhyay A., Aggarwal B.B. (2004). RKIP sensitizes prostate and breast cancer cells to drug-induced apoptosis. J. Biol. Chem..

[49] Yousuf S., Duan M., Moen E.L., Cross-Knorr S., Brilliant K., Bonavida B., LaValle T., Yeung K.C., Al-Mulla F., Chin E. (2014). Raf kinase inhibitor protein (RKIP) blocks signal transducer and activator of transcription 3 (STAT3) activation in breast and prostate cancer. PLoS ONE.

[50] Chen T.-C., Hsu Y.-L., Tsai Y.-C., Chang Y.-W., Kuo P.-L., Chen Y.-H. (2014). Gemifloxacin inhibits migration and invasion and induces mesenchymal-epithelial transition in human breast adenocarcinoma cells. J. Mol. Med. Berl. Ger..

[51] Beshir A.B., Argueta C.E., Menikarachchi L.C., Gascón J.A., Fenteany G. (2011). Locostatin Disrupts Association of Raf Kinase Inhibitor Protein with Binding Proteins by Modifying a Conserved Histidine Residue in the Ligand-Binding Pocket. Forum Immunopathol. Dis. Ther..

[52] Zhu S., Mc Henry K.T., Lane W.S., Fenteany G. (2005). A chemical inhibitor reveals the role of Raf kinase inhibitor protein in cell migration. Chem. Biol..

[53] Shemon A.N., Eves E.M., Clark M.C., Heil G., Granovsky A., Zeng L., Imamoto A., Koide S., Rosner M.R. (2009). Raf Kinase Inhibitory Protein protects cells against locostatin-mediated inhibition of migration. PLoS ONE.

[54] Zebisch A., Wölfler A., Fried I., Wolf O., Lind K., Bodner C., Haller M., Drasche A., Pirkebner D., Matallanas D. (2012). Frequent loss of RAF kinase inhibitor protein expression in acute myeloid leukemia. Leukemia.

[55] Caraffini V., Perfler B., Berg J.L., Uhl B., Schauer S., Kashofer K., Ghaffari-Tabrizi-Wizsy N., Strobl H., Wölfler A., Hoefler G. (2018). Loss of RKIP is a frequent event in myeloid sarcoma and promotes leukemic tissue infiltration. Blood.

[56] Stahl M., Kohrman N., Gore S.D., Kim T.K., Zeidan A.M., Prebet T. (2016). Epigenetics in Cancer: A Hematological Perspective. PLoS Genet..

[57] Huang H., Jiang X., Li Z., Li Y., Song C.-X., He C., Sun M., Chen P., Gurbuxani S., Wang J. (2013). TET1 plays an essential oncogenic role in MLL-rearranged leukemia. Proc. Natl. Acad. Sci. USA..

[58] Baritaki S., Huerta-Yepez S., da Lourdas Cabrava-Haimandez M., Sensi M., Canevari S., Libra M., Penichet M., Chen H., Berenson J.R., Bonavida B. (2011). Unique Pattern of Overexpression of Raf-1 Kinase Inhibitory Protein in Its Inactivated Phosphorylated Form in Human Multiple Myeloma. Forum Immunopathol. Dis. Ther..

[59] Jazirehi A.R., Vega M.I., Chatterjee D., Goodglick L., Bonavida B. (2004). Inhibition of the Raf-MEK1/2-ERK1/2 signaling pathway, Bcl-xL down-regulation, and chemosensitization of non-Hodgkin’s lymphoma B cells by Rituximab. Cancer Res..

[60] Vega M.I., Martínez-Paniagua M., Huerta-Yepez S., González-Bonilla C., Uematsu N., Bonavida B. (2009). Dysregulation of the cell survival/anti-apoptotic NF-κB pathway by the novel humanized BM-ca anti-CD20 mAb: Implication in chemosensitization. Int. J. Oncol..

[61] Baritaki S., Militello L., Malaponte G., Spandidos D.A., Salcedo M., Bonavida B. (2011). The anti-CD20 mAb LFB-R603 interrupts the dysregulated NF-κB/Snail/RKIP/PTEN resistance loop in B-NHL cells: Role in sensitization to TRAIL apoptosis. Int. J. Oncol..

[62] Trakul N., Menard R.E., Schade G.R., Qian Z., Rosner M.R. (2005). Raf kinase inhibitory protein regulates Raf-1 but not B-Raf kinase activation. J. Biol. Chem..

[63] Yeung K., Janosch P., McFerran B., Rose D.W., Mischak H., Sedivy J.M., Kolch W. (2000). Mechanism of suppression of the Raf/MEK/extracellular signal-regulated kinase pathway by the raf kinase inhibitor protein. Mol. Cell. Biol..

[64] Yeung K.C., Rose D.W., Dhillon A.S., Yaros D., Gustafsson M., Chatterjee D., McFerran B., Wyche J., Kolch W., Sedivy J.M. (2001). Raf kinase inhibitor protein interacts with NF-κB-inducing kinase and TAK1 and inhibits NF-κB activation. Mol. Cell. Biol..

[65] Grimaldi A.M., Simeone E., Festino L., Vanella V., Strudel M., Ascierto P.A. (2017). MEK Inhibitors in the Treatment of Metastatic Melanoma and Solid Tumors. Am. J. Clin. Dermatol..

[66] Battula V.L., Nguyen K., Sun J., Pitner M.K., Yuan B., Bartholomeusz C., Hail N., Andreeff M. (2017). IKK inhibition by BMS-345541 suppresses breast tumorigenesis and metastases by targeting GD2+ cancer stem cells. Oncotarget.

[67] Andela V.B., Schwarz E.M., Puzas J.E., O’Keefe R.J., Rosier R.N. (2000). Tumor Metastasis and the Reciprocal Regulation of Prometastatic and Antimetastatic Factors by Nuclear Factor κB. Cancer Res..

[68] Golubovskaya V.M. (2014). Targeting FAK in human cancer: From finding to first clinical trials. Front. Biosci. Landmark Ed..

[69] Araujo J., Logothetis C. (2010). Dasatinib: A potent SRC inhibitor in clinical development for the treatment of solid tumors. Cancer Treat. Rev..

[70] Duncan J.S., Whittle M.C., Nakamura K., Abell A.N., Midland A.A., Zawistowski J.S., Johnson N.L., Granger D.A., Jordan N.V., Darr D.B. (2012). Dynamic reprogramming of the kinome in response to targeted MEK inhibition in triple-negative breast cancer. Cell.

[71] Noy R., Pollard J.W. (2014). Tumor-associated macrophages: From mechanisms to therapy. Immunity.

[72] Ideker T., Dutkowski J., Hood L. (2011). Boosting signal-to-noise in complex biology: Prior knowledge is power. Cell.

